# In vivo quantification of the [^11^C]DASB binding in the normal canine brain using positron emission tomography

**DOI:** 10.1186/s12917-015-0622-3

**Published:** 2015-12-24

**Authors:** Olivia Taylor, Nick Van Laeken, Filip De Vos, Ingeborgh Polis, Tim Bosmans, Ingeborg Goethals, Rik Achten, Andre Dobbeleir, Eva Vandermeulen, Chris Baeken, Jimmy Saunders, Kathelijne Peremans

**Affiliations:** Department of Medical Imaging and Small Animal Orthopedics, Faculty of Veterinary Medicine, Ghent University, Salisburylaan 133, 9820 Merelbeke, Belgium; Laboratory of Radiopharmacy, Faculty of Pharmaceutical Sciences, Ghent University, Ottergemsesteenweg 460, 9000 Ghent, Belgium; Department of Medicine and Clinical Biology of Small Animals, Faculty of Veterinary Medicine, Ghent University, Salisburylaan 133, 9820 Merelbeke, Belgium; Department of Nuclear Medicine, Ghent University Hospital, Ghent University, De Pintelaan 185, 9000 Ghent, Belgium; Department of Radiology, Ghent University Hospital, Ghent University, De Pintelaan 185, 9000 Ghent, Belgium; Department of Psychiatry and Medical Psychology, Ghent University Hospital, Ghent University, De Pintelaan 185, 9000 Ghent, Belgium

**Keywords:** Dogs, [^11^C]DASB, Brain, PET, Serotonin transporter, Multilinear reference tissue model 2

## Abstract

**Background:**

[^11^C]-3-amino-4-(2-dimethylaminomethyl-phenylsulfanyl)-benzonitrile ([^11^C]DASB) is currently the mostly used radiotracer for positron emission tomography (PET) quantitative studies of the serotonin transporter (SERT) in the human brain but has never been validated in dogs. The first objective was therefore to evaluate normal [^11^C]DASB distribution in different brain regions of healthy dogs using PET. The second objective was to provide less invasive and more convenient alternative methods to the arterial sampling-based kinetic analysis.

**Results:**

A dynamic acquisition of the brain was performed during 90 min. The PET images were coregistered with the magnetic resonance images taken prior to the study in order to manually drawn 20 regions of interest (ROIs). The highest radioactivity concentration of [^11^C]DASB was observed in the hypothalamus, raphe nuclei and thalamus and lowest levels in the parietal cortex, occipital cortex and cerebellum.

The regional radioactivity in those 20 ROIs was quantified using the multilinear reference tissue model 2 (MRTM2) and a semi-quantitative method. The values showed least variability between 40 and 60 min and this time interval was set as the optimal time interval for [^11^C]DASB quantification in the canine brain. The correlation (R^2^) between the MRTM2 and the semi-quantitative method using the data between 40 and 60 min was 99.3 % (two-tailed p-value < 0.01).

**Conclusions:**

The reference tissue models and semi-quantitative method provide a more convenient alternative to invasive arterial sampling models in the evaluation of the SERT of the normal canine brain. The optimal time interval for static scanning is set at 40 to 60 min after tracer injection.

## Background

In vivo imaging of the living brain can be performed with positron emission tomography (PET), based on the detection of two opposite 511 keV gamma-rays that result from the annihilation of a positron and a negatron. The serotonergic system is one of the major neurotransmitter systems in the brain and is involved in a variety of neuropsychiatric disorders including anxiety disorders, schizophrenia, drug abuse, depression [1] Alzheimer and Parkinson’s diseases [2, 3]. Also in the dog, involvement of the serotonergic system in impulsive aggression, anxiety and compulsive disorders was demonstrated with single photon emission computed tomography [4–6]. Moreover, the serotonin enhancing drugs used to treat human disorders are used in canine behavior medicine [7–9].

As the primary molecular target of the selective serotonin reuptake inhibitors (SSRIs), the most common antidepressant drugs used in human medicine, the serotonin transporter (SERT) has been the focus of many studies in the past years. It is located on the presynaptic nerve endings of serotonergic neurons. It terminates neurotransmission by removing serotonin from the synaptic cleft (reuptake in the presynaptic neuron) and modulates thus the extracellular serotonin concentration [10].

Intensive research into development of suitable radiotracers to visualize and quantify this transporter with functional imaging modalities occurred in the past years in human (for review, [11]), but also in animal studies [12–15]. The [^11^C]-3-amino-4-(2-dimethylaminomethyl-phenylsulfanyl)-benzonitrile ([^11^C]DASB) is a recently developed highly selective radiotracer with a nanomolar affinity for the SERT for PET imaging [16]. Several studies in humans [17–21] showed that the regional distribution of the [^11^C]DASB was concordant with the known densities of SERT in the brain [22]. It is currently the most widely used radiotracer for PET quantitative studies of the SERT in human brain [2]. Up to now, [^11^C]DASB has been investigated in rodents [23], pigs [24], cats [12] and nonhuman primates [14], but never in dogs. As canine behavioral and human neuropsychiatric disorders, such as anxiety, aggressive and compulsive disorders, share many similarities [25], the dog represents a very interesting animal model for the investigation of human neuropsychiatric disorders. Furthermore, dogs represent a more practical and available alternative to other laboratory animals such as rodents or nonhuman primates. We already demonstrated in past studies that the investigation of the canine brain using radionuclides in general is feasible and that the results showed many similarities with human imaging studies [4–6, 15, 26–30].

The two parameters usually used to evaluate the [^11^C]DASB regional distribution are the total distribution volume (V_T_), representing the volume of tissue in which the radioligand would have to distribute to reach a concentration equal to that in the plasma [18], and the binding potential of the nondisplaceable compartment (BP_ND_), referring to the ratio at equilibrium of the specifically bound radioligand to that of the nondisplaceable radioligand in tissue [31]. The V_T_ values can be estimated using invasive kinetic methods, such as one-tissue compartment (1TC), two-tissue compartment (2TC) [32] and the linear graphical approach of Logan [33], requiring the placement of an arterial catheter. The V_T_ values can then be used to calculate indirectly binding potential values of the nondisplaceable compartment (BP_ND_) with the following equation [31]:

$$ B{P}_{ND}=\frac{V_T-{V}_{ND}}{V_{ND}}=\frac{V_T}{V_{ND}}-1 $$

where BP_ND_ = ratio at equilibrium of specifically bound (to receptor) radioligand to that of the nondisplaceable (free and non-specifically bound) radioligand in tissue; V_T_ = distribution volume of distribution of the total radioligand uptake in tissue in relation to total plasma concentration; V_ND_ = volume of distribution of the nondisplaceable radioligand concentration in relation to total plasma concentration [31]. Those BP_ND_ values can also be estimated by reference tissue methods, eliminating the need of arterial samples [19, 34, 35].

To the authors’ knowledge, this is the first study using PET imaging to evaluate SERT in the canine brain. The first objective was to evaluate regional distribution of the SERT in the normal canine brain with the tracer [^11^C]DASB. The second objective was to determine the equilibrium period (time interval during which regional tracer distribution is stable) in an attempt to use a semi-quantitative method to simplify the determination of the regional radiotracer uptake by using data from a static scan performed in that time interval. Furthermore, to correlate the regional tracer distribution values obtained with this semi-quantitative method with the data determined using a reference tissue method obtained from a dynamic scan.

## Methods

### Animals

Three healthy laboratory beagles and two purpose-bred laboratory dogs (four males and one female, 4 to 8 years old, weighting 11–32 kg) from the veterinary faculty of Ghent were enrolled. This prospective study was approved and conducted in accordance with the guidelines of the Ethical Committee of Ghent University (EC approval 2013/133).

The dogs were fasted for at least twelve hours before the scan. A 22G venous catheter was placed in one of the cephalic veins. The anesthetic protocol was similar for both the magnetic resonance imaging (MRI) and positron emission tomography/computed tomography (PET/CT) scan. They were premedicated with dexmedetomidin (Dexdomitor® 375 μg/m^2^ body surface area, IM). Anesthesia was induced with propofol (Propovet® 2-3 mg/kg depending on effect, IV) and, after endotracheal intubation, maintained with isoflurane (Isoflo® 1,2-1,4 % given to effect, administrated in 100 % oxygen via a circle system). A 22G arterial catheter was placed in one of the dorsal pedal arteries for the arterial sampling. Two anesthesiologists performed monitoring during and after anesthesia until the animals were fully awake.

### Imaging protocol

All five dogs underwent a MRI prior to the PET examination. Images were collected on a Siemens 3 T Magnetom Trio Tim system (Siemens Medical Systems, Erlangen, Germany) using a phased-array spine coil and a phased-array body matrix coil. Animals were anesthetized and placed head first in sternal recumbency in the scanner bore, with the front limbs extended caudally.

A structural scan was acquired using a T1-weighted 3D MPRAGE sequence with 176 sagittal slices. Following sequence parameters were used: TR = 2250 ms, TE = 4.18 ms, TI = 900 ms, parallel acquisition method = GRAPPA with acceleration factor = 2, matrix size = 256 × 256, sagittal, FOV = 220 mm, flip angle = 8°, voxel size = 0.9 × 0.86 × 0.86 mm^3^.

The [^11^C]DASB was synthetized by N-methylation of the precursor N-desmethyl-DASB with [^11^C]methyltriflate as previously described [36]. High chemical purities (>99 %) were obtained. The specific activity at the time of radiotracer injection was 43 ± 34 GBq/μmol.

Dogs were placed on the PET camera (Gemini PET/CT, Philips, Eindhoven, The Nederlands) in sternal recumbency with the front limbs extended caudally. A CT scan was performed for attenuation correction. The [^11^C]DASB was injected intravenously (mean activity ± SD: 7,8 ± 1,6 mCi or 289 ± 60 MBq) after and before a NaCl 0,9 % bolus. Data were acquired in list mode during a 90 min dynamic acquisition of the brain. The acquisition was started simultaneously to the injection of the radiotracer. The list mode data were reconstructed in 90 1-min frames for display purposes and determination of optimal scan time.

### Image analysis

A PET summation image of all frames and dynamic images were manually coregistered to each individual MR images using PMOD software (PMOD Technologies, Zurich, Switzerland).

**Fig. 1:**
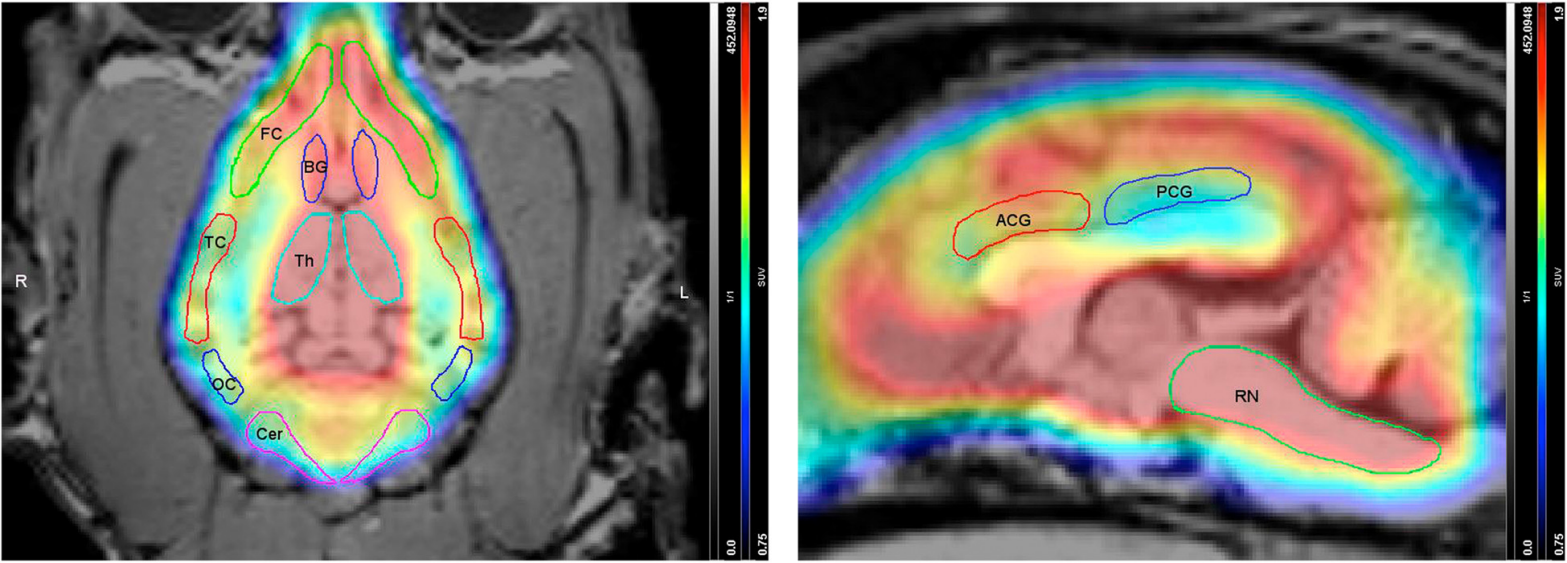
Regions of interest representative of the [^11^C]DASB distribution in the brain. Dorsal (left) and sagittal (right) sections of [^11^C]DASB PET images fused with the corresponding MRI images. The following regions are displayed: frontal cortex (FC), temporal cortex (TC), occipital cortex (OC), cerebellum (Cer), basal ganglia (BG), thalamus (Th), anterior cingulate cortex (ACG), posterior cingulate cortex (PCG) and the brainstem area where the raphe nuclei are located (RN)

Regions of interest (ROIs) were selected based on the known distribution of the SERT and drawn based on an anatomical atlas [37]. Twenty ROIs were manually delineated in the raphe nuclei, hypothalamus, thalamus (left and right), hippocampus (left and right), cerebellum (left and right), frontal cortex (left and right), parietal cortex (left and right), temporal cortex (left and right), occipital cortex (left and right), basal ganglia (left and right), anterior cingulate gyrus and posterior cingulate gyrus (Fig. 1). Within the cerebral cortex, only gray matter was included into the ROIs to measure the PET activity distribution. The ROIs were delineated on the dorsal plane T1-weighted images and extended to the co-registered PET images. Regional radioactivity was determined for each frame, corrected for decay and plotted versus time.

The effect of the scanning time on parameter’s stability was evaluated using the ratio analysis [18]. The ratios of five ROIs (raphe nuclei, thalamus, basal ganglia, frontal cortex and occipital cortex) over the cerebellum were determined for 20 min-scans starting every 10 min from 0 up to 70 min from the dynamic acquisition. The standard error of the ratio for each time interval was calculated to evaluate the stability of the parameters and therefore the optimal scanning time.

Several methods have been described for the quantification of [^11^C]DASB regional activity [18, 20, 32, 38]. In this article, we measured the BP_ND_ values using the multilinear reference tissue model 2 (MRTM2) and the cerebellum was chosen as the reference region, assuming the absence of SERT sites within this region [34]. As recommended by Meyer in his review paper [3], we selected the cerebellar cortex, excluding the vermis and keeping distance from white matter and venous sinuses. The values were calculated using the PMOD software (PMOD Technologies, Zurich, Switzerland). The nomenclature used in this article is the consensus recommended by Innis and coworkers [31].

In addition to the aforementioned quantitative compartmental analysis (MRTM2), a semi-quantitative method was also evaluated using the ratio of the concentration of radioactivity within the ROIs at the optimal scanning time (determined based on the standard error values), over the concentration of radioactivity within the reference region (cerebellum). This simple quantification method is preferred in clinical surroundings as scan duration is considerably reduced and, similar to the reference tissue models, does not require arterial sampling. These values were then compared with those from the MRTM2.

### Statistical analysis

To evaluate the usefulness of the semi-quantitative method, the coefficient of determination (R^2^) between [^11^C]DASB binding potential values in 5 different regions (raphe nuclei, thalamus, basal ganglia, frontal cortex and occipital cortex) obtained from the MRTM2 and the values obtained from the semi-quantitative ratio analysis was calculated to estimate the correlation between those two methods. Statistical analysis was performed using SPSS software (version 18.0; SPSS, Chicago, IL).

## Results

### Regional time-activity curves

**Fig. 2:**
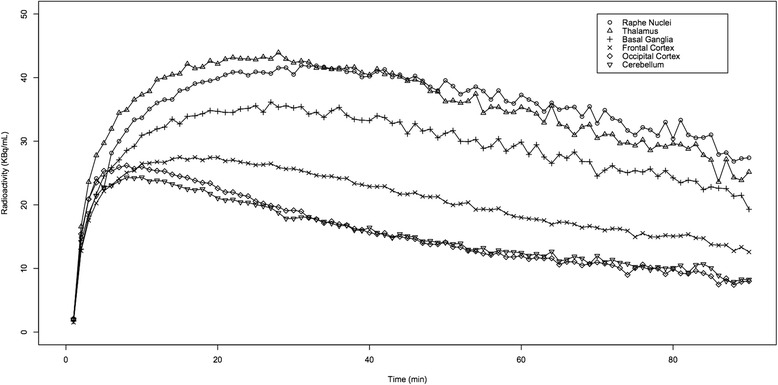
Time-activity curves for regional brain radioactivity following intravenous injection of [^11^C]DASB. Time-activity curves of six of the twenty regions of interest are displayed and represent the mean values of the five experiments. These six regions were chosen because they represent regions with high (raphe nuclei and thalamus), intermediate (basal ganglia and frontal cortex) and low (occipital cortex and cerebellum) radioactivity concentration. Except for the raphe nuclei (in the sagittal plane), the mean values of the right and left sides were taken for the five other regions

Regional time-activity curves (TACs) were obtained for each dog and each ROI. The highest radioactivity concentration of [^11^C]DASB was observed in the hypothalamus, raphe nuclei and thalamus, intermediate levels were observed in the basal ganglia, hippocampus and frontal cortex, modest levels in the anterior and posterior cingulate gyri and temporal cortex, and low levels in the parietal cortex, occipital cortex and cerebellum. The TACs of six representative regions of different radioactivity levels, high (raphe nuclei and thalamus), intermediate (basal ganglia and frontal cortex) and low (occipital cortex and cerebellum), are displayed in Fig. 2.

### Optimal static scanning time

**Fig. 3:**
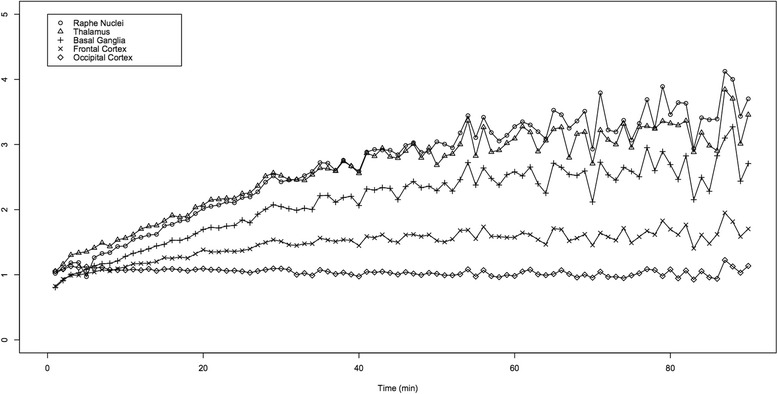
Time curves of the ratio of five representative ROIs over the cerebellum. These curves demonstrate that the values reach a plateau after approximately 40 min and become more variable after approximately 70 min of scanning. The optimal scanning time was determined from 40 to 60 min. The values correspond to the mean of the five experiments for five representative ROIs (raphe nuclei, thalamus, basal ganglia, frontal cortex and occipital cortex). Except for the raphe nuclei (in the sagittal plane), the mean values of the right and left sides were taken for the four other regions

Time curves of the ratios of radioactivity of five ROIs (raphe nuclei, thalamus, basal ganglia, frontal cortex and occipital cortex) over the cerebellum are displayed in Fig. 3. These curves demonstrate that the values reach a plateau after approximately 40 min and become more variable after approximately 60 min of scanning due to the decay of ^11^C.

The ratios of the same five ROIs representing regions of high (raphe nuclei and thalamus), moderate (basal ganglia and frontal cortex) and low (occipital cortex) SERT densities over the cerebellum were calculated for 20 min-scans starting every 10 min from the dynamic data. The standard error was determined in order to assess the stability of the parameters and the optimal scanning time (Table 1). The values were demonstrated to be less variable from 30 to 70 min, the standard error values being 2.6 % when scanning from 30 to 50 min, 2.5 % from 40 to 60 min and 2.9 % from 50 to 70 min. Therefore, the optimal scanning time was determined to be between 40 to 60 min, as the standard error of the values obtained during this time interval was the lowest.

**Table 1:** Ratio of five ROIs over the cerebellum for 20 min-scans

Scanning time (min)	Raphe nuclei	Thalamus	Basal ganglia	Frontal cortex	Occipital cortex	Mean SE
0 to 20	1,51 ± 0,07	1,64 ± 0,06	1,32 ± 0,05	1,15 ± 0,03	1,09 ± 0,005	0.045
10 to 30	1,93 ± 0,06	2,02 ± 0,06	1,63 ± 0,05	1,31 ± 0,02	1,07 ± 0,003	0.042
20 to 40	2,37 ± 0,05	2,40 ± 0,04	1,95 ± 0,04	1,45 ± 0,02	1,05 ± 0,01	0.031
30 to 50	2,73 ± 0,04	2,69 ± 0,04	2,19 ± 0,03	1,53 ± 0,01	1,03 ± 0,01	0.026
40 to 60	3,00 ± 0,04	2,89 ± 0,03	2,36 ± 0,03	1,57 ± 0,01	1,01 ± 0,01	0.025
50 to 70	3,19 ± 0,04	2,99 ± 0,04	2,45 ± 0,03	1,57 ± 0,02	1,00 ± 0,01	0.029
60 to 80	3,32 ± 0,05	3,10 ± 0,04	2,55 ± 0,04	1,59 ± 0,02	1,00 ± 0,01	0.034
70 to 90	3,20 ± 0,07	3,03 ± 0,05	2,49 ± 0,06	1,58 ± 0,03	1,02 ± 0,02	0.046

The values of the five ROIs correspond to the mean values of the five experiments ± standard error. Except for the raphe nuclei (in the sagittal plane), the mean values of the right and left sides were taken for the four other regions

The standard error (SE) was the lowest for the values between 40 and 60 min of scanning, which was therefore determined as the optimal time interval

### Quantification of [^11^C]DASB regional activity

The BP_ND_ values were derived from the reference tissue model MRTM2 using the cerebellum as a reference region (Table 2). These values were obtained for 18 ROIs (raphe nuclei, hypothalamus, left and right thalamus, left and right hippocampus, left and right frontal cortex, left and right parietal cortex, left and right temporal cortex, left and right occipital cortex, left and right basal ganglia, anterior cingulate gyrus, posterior cingulate gyrus), the last two ROIs over the left and right cerebellum being used as the reference region.

**Table 2:** BP_ND_ values obtained from MRTM2

	MRTM2
Region	BPND
Raphe nuclei	1,80 ± 0,37
Hypothalamus	1,75 ± 0,34
Hippocampus L	0,93 ± 0,43
Hippocampus R	0,85 ± 0,18
Thalamus L	1,75 ± 0,60
Thalamus R	1,70 ± 0,40
Frontal cortex R	0,50 ± 0,17
Frontal cortex L	0,50 ± 0,23
Basal ganglia R	1,15 ± 0,49
Basal ganglia L	1,27 ± 0,77
ACG	0,39 ± 0,26
PCG	0,25 ± 0,17
Temporal cortex R	0,26 ± 0,11
Temporal cortex L	0,28 ± 0,19
Occipital cortex R	0,05 ± 0,11
Occipital cortex L	0,05 ± 0,10
Parietal cortex R	0,17 ± 0,17
Parietal cortex L	0,20 ± 0,18

The BP_ND_ values for the MRTM2 method were automatically calculated using PMOD software and correspond to the mean ± standard deviation of the five experiments for 18 ROIs (raphe nuclei, hypothalamus, left and right thalamus, left and right hippocampus, left and right frontal cortex, left and right parietal cortex, left and right temporal cortex, left and right occipital cortex, left and right basal ganglia, anterior cingulate gyrus, posterior cingulate gyrus). The ROIs over the left and right cerebellum were used as the reference region

*BP* binding potential of the nondisplaceable compartment, *L* left, *R* right

For the MRTM2, the k’_2_ value was calculated using the Multilinear Reference Tissue Model (MRTM) as the mean k_2_ (clearance rate constant from a region of interest to plasma) value of five high-SERT binding regions: raphe nuclei, right and left thalamus, right and left basal ganglia. The mean value of the k’_2_ parameter for the five experiments was 0.096 ± 0.013.

Given the results of the aforementioned scanning time interval analysis, the semi-quantitative analysis was performed using the data between 40 to 60 min. The ratios of the concentration of radioactivity within the same 18 ROIs over the cerebellum are presented in Table 3.

**Table 3:** Semi-quantitative ratio analysis

Region	Ratio	Region	Ratio	Region	Ratio
Raphe nuclei	3,03 ± 0,51	Frontal cortex R	1,59 ± 0,25	Temporal cortex R	1,25 ± 0,20
Hypothalamus	2,85 ± 0,55	Frontal cortex L	1,57 ± 0,29	Temporal cortex L	1,27 ± 0,31
Hippocampus L	2,07 ± 0,44	Basal ganglia R	2,13 ± 0,74	Occipital cortex R	1,32 ± 0,52
Hippocampus R	2,05 ± 0,30	Basal ganglia L	2,22 ± 0,94	Occipital cortex L	1,29 ± 0,45
Thalamus L	2,91 ± 0,75	ACG	1,41 ± 0,38	Parietal cortex R	1,17 ± 0,21
Thalamus R	2,91 ± 0,54	PCG	1,22 ± 0,24	Parietal cortex L	1,21 ± 0,23

Semi-quantitative values were obtained using the ratio analysis method: ratio of the concentration of radioactivity within the same 18 ROIs as for MRTM2 (Table 2) over the concentration of radioactivity within the cerebellum, using the data from 40 to 60 min

The values correspond to the mean ± standard deviation of the five experiments

*Ratio* ROI/cerebellum, *L* left; *R* right

### Statistical analysis: comparison of methods

**Fig. 4:**
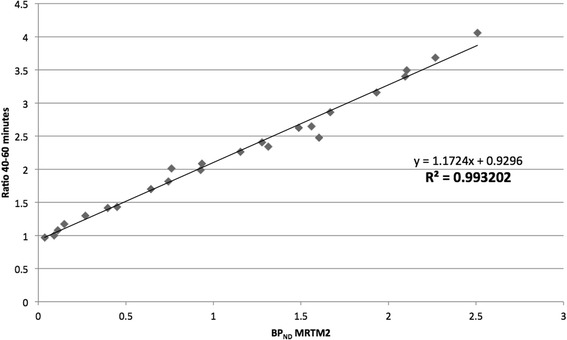
Correlation between the semi-quantitative ratio analysis and the reference tissue model MRTM2. The coefficient of determination (R^2^) between the BP_ND_ values obtained from the MRTM2 and the values obtained from the semi-quantitative analysis was determined. The values correspond to the five experiments for five ROIs over representative regions of high (raphe nuclei and thalamus), intermediate (basal ganglia and frontal cortex) and low (occipital cortex) radioactivity concentration. Except for the raphe nuclei (in the sagittal plane), the mean values of the right and left sides were taken for the four other regions. BP_ND_ = binding potential of the nondisplaceable compartment

The correlation (R^2^) between the values obtained from the reference tissue model MRTM2 and the semi-quantitative ratio analysis using the data between 40 and 60 min was 99.3 % (two-tailed p-value < 0.01), and therefore statistically significant (Fig. 4).

## Discussion

The primary objective of this study was to evaluate the SERT density in the normal canine brain from analysis of [^11^C]DASB regional distribution using PET imaging. The highest radioactivity levels were observed in the hypothalamus, raphe nuclei and thalamus, intermediate levels were observed in the basal ganglia, hippocampus and frontal cortex, modest levels in the anterior and posterior cingulate gyri and temporal cortex, and low levels in the parietal cortex, occipital cortex and cerebellum. These results are consistent with the previous human [17–21, 39] and animal [12, 14] studies.

As previously mentioned, the two parameters V_T_ and BP used to evaluate the [^11^C]DASB regional distribution can be estimated using invasive and time-consuming kinetic methods such as 1TC, 2TC and Logan graphical models which require the placement of an arterial catheter. BP values can also be estimated by reference tissue methods that represent a noninvasive alternative method to evaluate the SERT density of the canine brain, withdrawing the need of arterial samples. A previous study compared the BP_ND_ values obtained via a 2TC model with those calculated using several reference tissue models, the simplified reference tissue model 2, the MRTM2 and the Logan noninvasive model. The MRTM2 model demonstrated the highest correlation (R^2^ = 0.96) with the 2TC, and was therefore chosen as reference tissue method of choice in our study [40].

To perform the reference tissue methods, the cerebellar cortex is selected as reference region. The properties required for an ideal reference region are similar free and nonspecific tracer binding properties as compared to regions with specific binding, and negligible specific binding of the radiotracer. Previously, the white matter and occipital cortex were also tested as reference regions. Although having very low levels of SERT, the possible difference of pharmacokinetics in free and nonspecific compartments between white matter and gray matter, caused the cerebellar cortex to become the more commonly used reference region, even though it is not completely devoid of SERT binding sites [41].

The second objective was to investigate the possibility of replacing the dynamic scan by a static acquisition. The influence of the scanning time was evaluated in order to assess whether it would be possible to perform a shorter (20 min) static acquisition instead of the 90 min dynamic acquisition for SERT binding estimations in patients. Our results demonstrated that the values show least variability between 40 and 60 min. In human studies, ratios of radioactivity within the ROIs to that in the cerebellum were calculated using data from 55 to 90 min after injection of the radiotracer [17, 18]. In our study, the ratio values were the most variable in the late acquisition, from 70 to 90 min (SE = 4.6 %) despite relative higher injected activity compared to humans. The explanation could be the increase in noise towards the end of the scan because of the physical decay of the radionuclide (T_1/2_ of ^11^C = 20 min) [38]. For these reasons, it has been previously established in human studies that there is no need to scan longer than approximately 90 min [18, 20, 38].

Finally, a semi-quantitative analysis was performed by measuring the ratio of the radioactivity within several ROIs to the radioactivity within the cerebellum, using the data from 40 to 60 min. This method correlated significantly to the MRTM2 (R^2^ = 0.993). Therefore, a 20 min static acquisition performed 40 min after intravenous injection of [^11^C]DASB represents another more convenient and faster alternative method to evaluate the SERT of the canine brain in clinical surroundings.

The presence of [^11^C]DASB radiometabolites in the brain and their potential interference with the measurements cannot be excluded. Nevertheless, [^11^C]DASB is a highly selective radiotracer with a high affinity for the SERT [16]. Its radiometabolites are mainly polar and therefore unlikely to cross the blood–brain barrier and interfere with the measurements [17, 42].

## Conclusions

Evaluation of the SERT in the canine brain can be performed after administration of [^11^C]DASB using PET imaging. Given the similarities between canine behavioral disorders and human neuropsychiatric disorders, the dog could represent a valuable model for research about the involvement of the SERT in neuropsychiatry. Reference tissue methods based on a dynamic scan and the semi-quantitative ratio method based on a static scan provide less invasive and more convenient alternatives to the arterial sampling based kinetic analysis.

## Abbreviations

[^11^C]DASB: [^11^C]-3-amino-4-(2-dimethylaminomethyl-phenylsulfanyl)-benzonitrile
1TC: one-tissue compartment
2TC: two-tissue compartment
BP_ND_: binding potential of the nondisplaceable compartment
MRI: magnetic resonance imaging
MRTM2: multilinear reference tissue model 2
PET: positron emission tomography
PET/CT: positron emission tomography/computed tomography
ROIs: regions of interest
SERT: serotonin transporter
SSRIs: selective serotonin reuptake inhibitors
TACs: time-activity curves
V_ND_: volume of distribution of the nondisplaceable compartment
V_T_: total distribution volume
